# Overcoming cancer drug resistance: insights into Apoptotic pathway modulation by plant-based nanoparticle and natural products

**DOI:** 10.3389/fonc.2025.1677380

**Published:** 2025-10-21

**Authors:** Amit Kumar Singh, Prem Prakash Kushwaha, Ajaya Kumar Singh, Abhay K. Pandey

**Affiliations:** ^1^ Department of Botany, BMK Govt. Girls College, Balod, India; ^2^ Department of Biological, Geological, and Environmental Sciences, Cleveland State University, Cleveland, OH, United States; ^3^ Departments of Chemistry, Government VYT PG Autonomous College, Durg, India; ^4^ Departmentsof Biochemistry, University of Allahabad, Prayagraj, India

**Keywords:** apoptosis, natural products, drug resistance, cancer, nanoparticle

## Abstract

Cancer is a complex progressive disease, characterized by uncontrolled cell growth, posing a serious global health problem across the population. The traditional treatments for this disease include chemotherapy, surgery, and radiation therapy, forming the backbone of care. However, over time, tumor cells often develop resistance to these treatments, making drug resistance a leading factor in disease progression and poor clinical outcomes for some patients. Cancer drug resistance is multifaceted at both the molecular and cellular levels. An important mechanism through which cancer cells acquire resistance to multiple drugs is dysregulated apoptosis (programmed cell death). This compromised apoptotic pathway resulted in prolongs cancer cell survival, accumulation of mutations that promote angiogenesis, stimulation of cell proliferation, impaired differentiation, and enhanced invasiveness during tumor progression. In the past few years, plant-derived natural products have garnered attention as promising therapeutic agents against drug-resistant cancers due to their minimal side effects and potent anticancer properties. However, their clinical application faces several challenges, including poor solubility, limited absorption, restricted tissue distribution, and rapid metabolism. An effective approach to address these limitations involves utilizing nanoparticles and nanomaterials, which can improve pharmacokinetics, enhance tumor-specific targeting, minimize side effects, and overcome drug resistance. This review delves into the fundamental molecular pathways associated with apoptosis and explores how phytochemicals and plant extracts, in combination with conventional drugs and plant-based nanoparticles can be utilized to treat cancer as well as cancer drug resistance by modulating its programmed cell death network.

## 1 Introduction

Cancer is a multistage, complex disease typically classified into six phases of initiation, promotion, transformation, progression, invasion, and metastasis. It is characterized by the abnormal proliferation of cells in different tissues. Cellular proliferation is usually caused by mutations and the deregulation of genes that control cell division and growth (1–3). In general, cancer remains a major health issue worldwide, encompassing a diverse group of diseases that affect all populations. Therefore, low- and middle-income countries face significant challenges in addressing the burden of the disease while resources are limited, which contributes to higher mortality rates (4). For instance, in India, it is estimated that 70% of cancer deaths occur within the first year of diagnosis; these causes include late presentation and fewer options of quality health care (5). Furthermore, while the global cancer incidence typically increases with higher life expectancy and living standards, certain cancers, like cervical cancer, disproportionately affect populations with lower socioeconomic metrics. Recent global statistics have estimated that nearly 20 million new cancer cases and 9.7 million associated deaths occurred in 2022. However, these values are likely to increase as the annual estimates for cases and deaths reach 29.9 million and 15.3 million by 2040, respectively (6–8). In India, mortality due to cancer mainly targets those between 30–69 years of age; while around 15% cases have children and adolescents as victims, which is again much higher than the global estimate of 0.5% (5).

Traditional treatments for cancer include chemotherapy, surgery, and radiation therapy. These treatments aim to eliminate cancer cells but, in the process, harm healthy tissues with adverse effects. Multidrug resistance (MDR) is a challenge in chemotherapy because of gene mutations, overexpression of resistance-related proteins, and mechanisms inhibiting drug efficacy. Repeated chemotherapy cycles exacerbate MDR, reducing the effectiveness of anticancer agents and impeding treatment success (4, 9).

Apoptosis or the programmed cell death mechanism holds the key to maintaining cellular homeostasis as well as preventing malignancies (10). There are two major pathways for apoptosis: intrinsic (mitochondrial) and extrinsic (death receptor), which depend on the nature of the activating signals. Dysregulation of these pathways has dire implications, especially in cancer, where the intrinsic pathway is often restrained. Inhibition of this pathway allows cancer cells to avoid programmed cell death, live longer, and accrue mutations that eventually cause uncontrolled proliferation, progressive tumor advancement, and therapy resistance (1, 11–13). In recent times, phytomedicines have been viewed as potential alternatives to conventional therapies. They exhibit anticancer properties with relatively fewer side effects. However, solubility issues, poor permeability, and lowered bioavailability stand as barriers to their use in the clinical realm. Nanotechnology-based delivery systems have thus emerged as a potent solution towards ameliorating these limitations; they enhance the bioavailability, stability, and targeted delivery of these phytochemicals. Advanced formulations through these system result in controlled release and offer protection to active compounds from degradation, thereby providing an effective yet less toxic therapeutic approach (4, 12–14).

This review examines recent advances in phytochemical-based nanoparticles that modulate apoptotic pathways to inhibit cancer progression and overcome cancer drug resistance, while also providing insights into ongoing preclinical and clinical studies with emphasis on translational challenges and future opportunities for clinical application.

## 2 Cancer drug resistance

**Figure 1:**
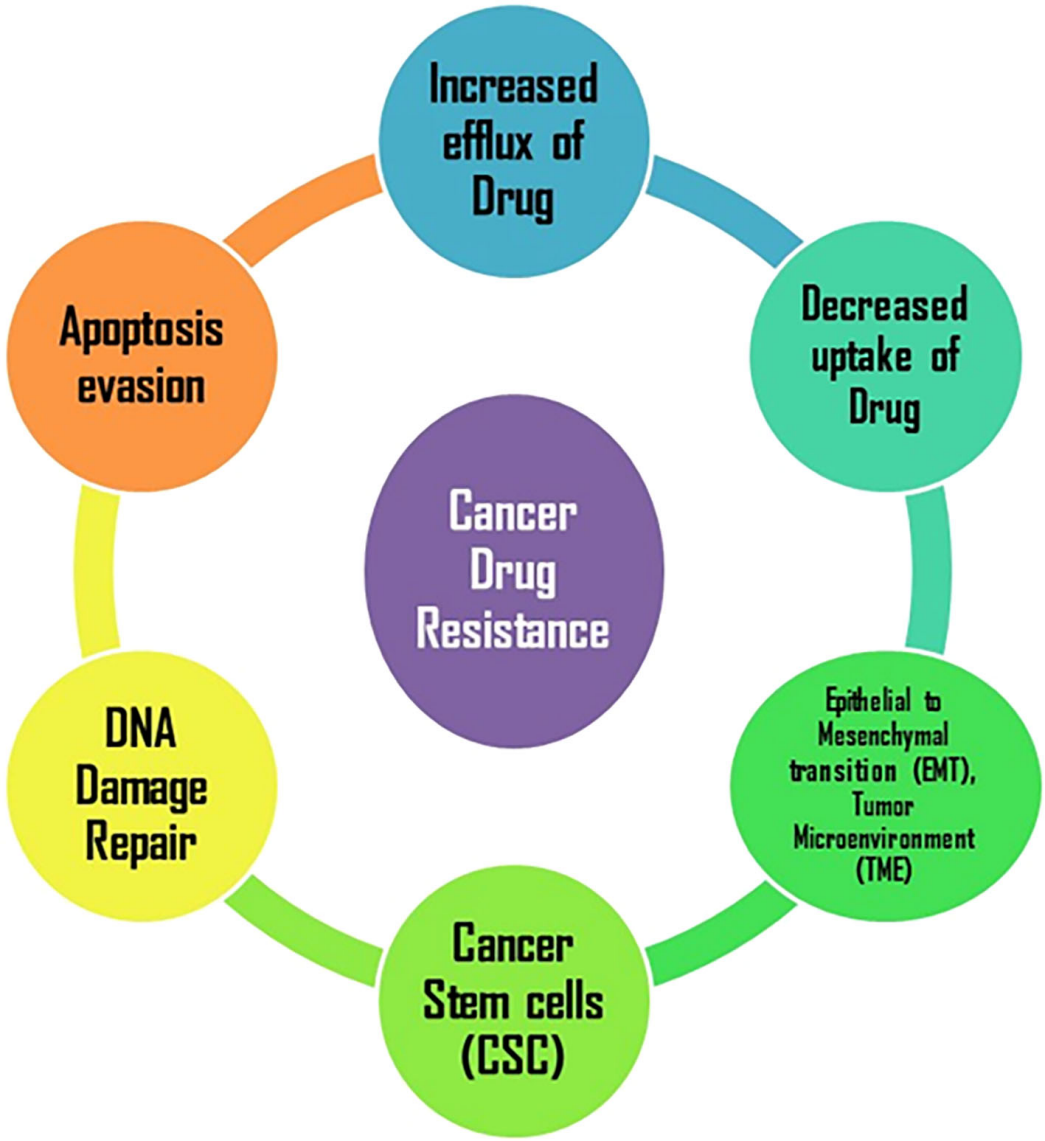
Major mechanism/pathway responsible for cancer drug resistance.

Drug resistance generally presents one of the major obstacles in oncology, usually mitigating benefits in terms of treatment efficacy and promoting disease recurrence. The mechanisms behind drug resistance include efflux of drugs, modification of target proteins, increased DNA repair, and evocation of apoptosis as depicted in **Figure 1**. Cancer cells are thought to have an active mechanism that removes and expels chemotherapeutic drugs away from the cell, which results in reduced intracellular concentration and decreased efficacy of therapy. ATP-binding cassette transporters, such as P-glycoprotein, represent a major mechanism that allows cancer cells to tolerate higher drug concentrations than normal cells. The other major route is modifications of the targets, especially targeted therapies. Mutations in the drug-target proteins both reduced the activity of the BCR-ABL gene in chronic myeloid leukemia and decreased the binding affinity of the inhibitors, including imatinib, thereby reducing their effectiveness. This is also through enhanced DNA repair, in that most therapies kill cells through the induction of DNA damage. Cancer cells resist this by upregulating repair pathways, like homologous recombination, to rapidly fix the damage and lend support to proliferation. For instance, BRCA mutations make tumors initially sensitive to therapies that attack the DNA repair pathways but lead to resistance with the reactivation of these pathways. Second, most therapeutic agents target the apoptosis pathway, but cancer cells often evade this process. It can occur through the overexpression of anti-apoptotic proteins, such as Bcl-2, or downregulation of pro-apoptotic proteins and thus allow cells to survive even in the presence of treatment. In summary, these adaptive strategies together advance the idea of resilience in cancer cells and urge innovative treatments, such as combination therapy, to overcome resistance and create better outcomes from treatment (9, 13, 15).

## 3 Apoptotic signaling pathways in cancer cells

Cancer treatment strategies often focus on to attain or at least halt the uncontrolled proliferation of cancer cells. It is, in fact, highly effective to exploit its own programmed cell death mechanism (10). There are two types of apoptosis pathways leading to apoptosis; extrinsic and intrinsic activated by extracellular and intracellular signals respectively.

### 3.1 Intrinsic pathway

The intrinsic pathway of apoptosis is caused by a range of physical and chemical stimuli with hypoxia, deprivation of growth factors, and other types of cellular stimuli including excess calcium ions (Ca^2+^), cellular detachment, and many stress signals including DNA damage, oncogene activation, oxidative stress, and irradiation (10, 11). This is essentially a course through mitochondria-dependent and operates around mitochondrial proteins.

**Figure 2:**
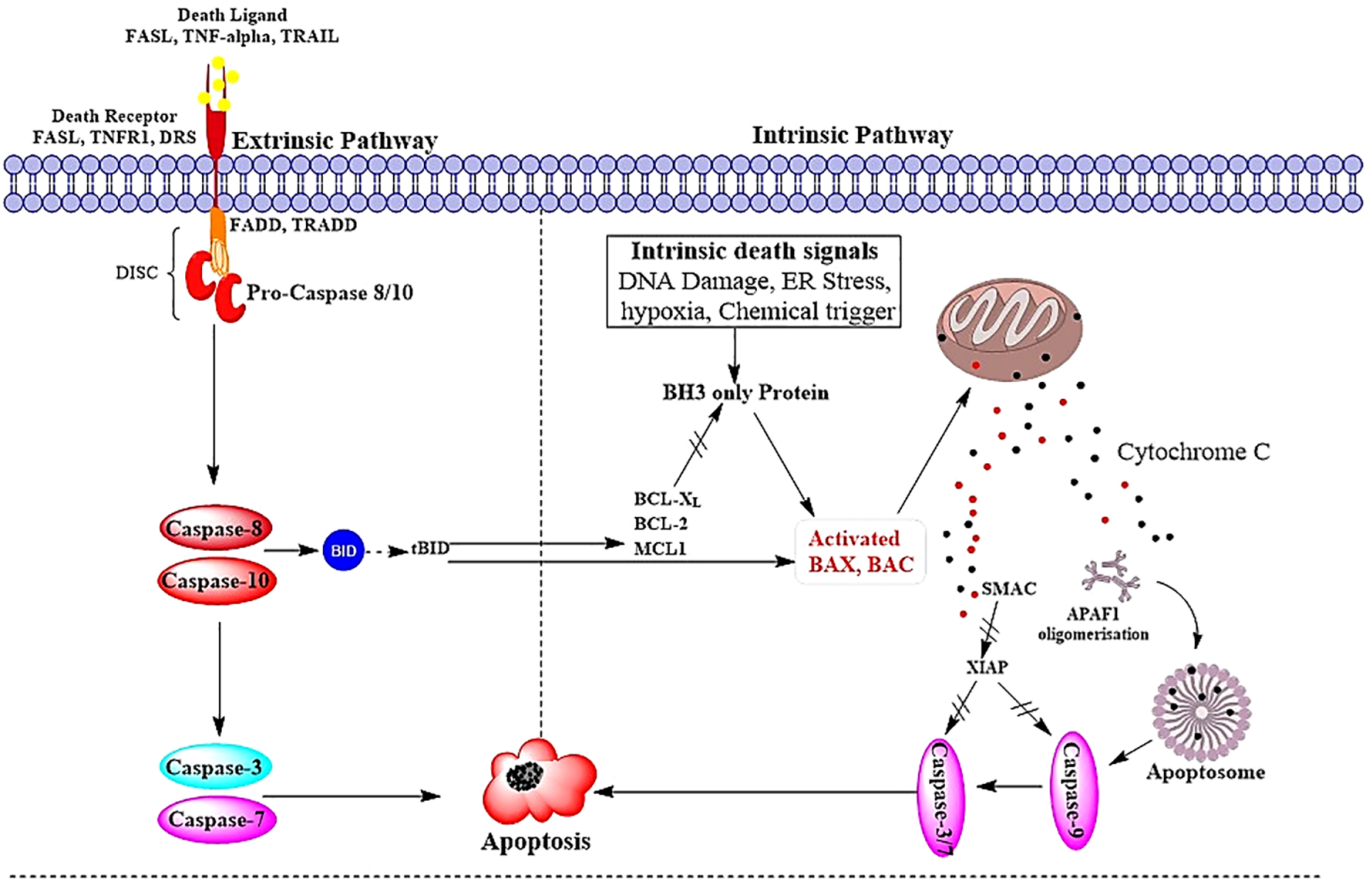
Mechanism of extrinsic and intrinsic pathway of Apoptosis. FASL, FS-7- associated surface antigen ligand; TRAIL, Targeting TNF-related apoptosis-inducing ligand; TNFR1, Tumor necrosis factor receptor 1; TNF-α, Tumor necrosis factor-alpha; DRS, Death receptors; DISC, death-inducing signaling complex; BID, BH3 interacting-domain; MCL, myeloid leukemia cell differentiation protein; BAX, Bcl-2-associated X protein; BAK, B-cell lymphoma 2 killer; SMAC, Second mitochondria-derived activator of caspase; XIAP, X-linked inhibitor of apoptosis protein; MOMP, mitochondrial outer membrane permeabilization; and APAF, Apoptotic protease activating factor; Adapted from (12).

This intrinsic pathway starts with the upregulation of BH3-only proteins leading to the attachment of pro-apoptotic proteins (Bax, Bak) into the mitochondrial membrane through their oligomerization triggers mitochondrial outer membrane permeabilization (MOMP), and subsequently, the liberation of cytochrome c from the intermembrane space into the cytosol. However, this process is regulated by anti-apoptotic proteins, Bcl-2 and Bcl-xL, whose functions are to maintain cellular homeostasis (**Figure 2**) (11, 16, 17). As soon as it reaches to cytoplasm, there is an association between cytochrome c with apoptotic protease-activating factor-1 (APAF-1), procaspase-9, and dATP resulted in the formation of apoptosome complex. Inside the apoptosome, there is a conversion of procaspase-9 to caspase-9 which further converts executioner caspases-3 and -7 into their active forms. These killer caspases degrade cellular proteins very quickly; hence, there is programmed cell death (**Figure 2**) (11, 18).

### 3.2 Extrinsic pathway

The extrinsic pathway is referred to as the death receptor-dependent pathway, which plays a vital role in cellular homeostasis and importantly modulates immune responses within the body. The pathway starts with the activation of death receptors through several on the surface of the cell including Fas ligand (Fas-L), TNF-related apoptosis-inducing ligand (TRAIL), and tumor necrosis factor (TNF). Once these receptors are activated, adaptor proteins i.e., TNF receptor-associated death domain (TRADD and Fas-associated death domain (FADD) are recruited onto the activated death receptor where a cascade will lead to apoptosis (**Figure 2**) (11, 19).

One of the most significant and crucial steps in this very complex process involves the high levels of interaction between caspase-8 and FADD that come together to form what is termed the death-inducing signaling complex, more frequently referred to as DISC. The DISC is an important complex because it causes the activation of the critical procaspases-8 and -10, which ultimately leads to the activation of the so-called executioner caspases, specifically caspases-3, -6, and -7 (**Figure 2**) (10, 11). The activation of these executioner caspases is critical because they then go on to cleave many cellular proteins as well as destroy the cytoskeleton resulting in the process termed apoptosis. Regulation of DISC activity is critical and is mediated by a protein known as c-FLIP, which serves as a moderator inhibitor. The protein is actually similar in structure to caspase-8 but lacks any enzymatic activity (20).

## 4 Targeting apoptotic pathways to overcome drug resistance

Anti-cancer drugs are designed specifically to address the crucial challenge of selectively eliminating cancerous cells while at the same time attempting to minimize any potential side effects that may affect healthy cells. The treatments invented against cancer are mainly multi-faceted amalgams used today that normally comprise surgery, radiation, and several types of drugs that contain chemotherapeutic drugs in this list. These drugs are important since they can alleviate symptoms of cancer, prolong life, and occasionally make the patient go into a state of complete remission whereby the disease cannot be traced. Ideally, a successful cancer treatment should be so strong that it would reflect minimal damage or destruction to healthy cells and simultaneously actively attack and destroy cancerous cell (1, 9, 11, 13).

A crucial aspect affecting both the progression of cancer and the effectiveness of treatments is apoptosis, related to maintaining cellular equilibrium. The initial stages of research thought that, increased cellular proliferation was thought to solely result from the accumulation of new cells. However, it is now understood that reduced cell death significantly contributes to this complex process of cancer growth. Cancer cells are characterized by their evasion to apoptosis, so this is an important focus for efforts to help overcome drug resistance. Cancer cells often show overexpression of anti-apoptotic proteins, like Bcl-2 and Bcl-xl, which are critical for evading the normal process of programmed cell death. Inhibition of such specific proteins can reactivate the intrinsic apoptotic pathway, thereby significantly sensitizing the cancer cells towards drug induced apoptosis. Besides this, mutations in p53; a critical tumor suppressor protein can remarkably impair its pro-apoptotic functions, thereby further promoting survival and persistence of cancer cells in the body. Other pathways hijacked by cancer cells include the autophagy-apoptosis crosstalk, death receptor pathway, and the activation of inhibitor of apoptosis proteins (IAPs) like XIAP, cIAP1, and cIAP2, amongst others to prevent apoptosis (21, 22).

Given these complexities, the scientific world is facing greater urgency in finding natural products that can act as good sources of new apoptotic inducers. Natural compounds, either in crude extract form or in isolated secondary metabolite form plants, marine organisms, or animals, have been demonstrated to bear considerable potential both in the prevention and treatment of cancer. Mechanistic studies of these compounds through various researches provide a deeper understanding of their molecular action, paving the way for the development of future therapeutic agents to combat cancer drug resistance (1, 9, 12, 23).

## 5 Plant extract/derived natural bioactive compound targeting apoptotic and associated pathway of cancer cells

Resistance to chemotherapy and radiotherapy along with the toxicity associated with these conventional treatment modalities emphasizes the immense need for anticancer strategies that would be not only safer but more effective. Traditional chemotherapeutic agents often lack specificity, targeting both healthy and tumor cells indiscriminately. However, ideal therapy should be able to selectively identify the normal cells from the cancerous ones, thereby minimizing collateral damage (11, 24).

Plant-derived compounds, known for their non-toxic nature, offer a promising alternative in this regard. Historically, plants are being used medicinally for over 5000 years, contributing greatly to modern medicine, with almost 25% of current pharmaceuticals derived directly or indirectly from plant sources. Secondary metabolites from plants are recognized as cost-effective, safe, and potent natural compounds with diverse bioactivities, including anticancer properties. These compounds are not essential for growth and development of plants, and depending on their biosynthetic pathways, categorized into classes like terpenoids, flavonoids, phenolics, and alkaloids. Ex. vincristine, camptothecin, vinblastine, resveratrol, curcumin, apigenin, gamma-tocopherol and lycopene etc. (25). These molecules have the unique characteristic of acting alone or synergistically to effectively target cancer hallmarks such as inhibition of specific cell cycle proteins, disrupting microtubule assembly, blocking angiogenesis, as well as the induction of apoptosis. Other than that, by actively repressing the cancer cell ability to inhibit pro-apoptotic proteins, these active phytochemicals notably enhance the susceptibility of cancerous cells against different treatments, such as ionizing radiation and chemotherapy, resulting in better therapeutic outcomes (25, 26).

Apoptosis is a highly regulated process of cell death that can play a crucial role in both development and the maintenance of homeostasis within the body. This important mechanism has emerged as a central focus in the field of cancer therapy. Unlike conventional cytotoxic agents, which damage otherwise healthy cells to kill cancer cells, apoptosis inducers target and kill exclusively those cells. They capitalize on the dysregulated machinery of apoptosis, commonly found inside tumors, to act in a more targeted manner. Incorporating natural compounds as cytotoxic or anti-carcinogenic agents represents a promising avenue for cancer prevention and therapy (27). Extensive research on the secondary metabolites derived from a wide array of natural sources provides valuable insights into the molecular mechanisms by which these compounds work, laying groundwork for formulating and creating highly effective therapeutic agents that can be used in the on-going battle against cancer (12, 13).

Graviola, a fruit-bearing tree, has long been recognized in alternative and traditional medicine for its therapeutic potential, particularly its anticancer properties (11, 28). It is known to inhibit BCL-2 proteins while enhancing BAX expression, ultimately promoting apoptosis. Although its precise mechanism remains unclear, graviola’s selective toxicity against cancer cells while sparing healthy ones makes it a promising alternative to conventional therapies like chemotherapy and radiotherapy (29). Beyond Graviola, numerous plant-derived compounds exhibit apoptotic activity against cancer cells. Black cohosh from *Actaearacemosa (* 30), juglone from *Juglans mandshurica (* 31), and genistein (32) are among the notable examples (11). Quercetin, abundant in apples and red onions, activates caspases to initiate apoptosis (32), while epigallocatechin-3-gallate (EGCG) from green tea and aloe-emodin from *Rheum palmatum* also demonstrate caspase-mediated apoptotic effects (11, 33).

Gallic acid (GA) and methyl gallate (MG) isolated from *Quercus infectoria* against HeLa cervical cancer cells. Both compounds exhibited strong antiproliferative activity with IC50 values of 10.00 ± 0.67 µg/mL (GA) and 11.00 ± 0.58 µg/mL (MG). AO/PI staining and Annexin-V FITC assays confirmed apoptotic cell death, with cell cycle arrest at the sub-G1 phase. Mechanistic studies revealed upregulation of p53 and Bax, downregulation of Bcl-2, and activation of caspases 8 and 9. These findings demonstrate that GA and MG inhibit cervical cancer cell growth by inducing apoptosis via both intrinsic and extrinsic pathways (1, 34). The methanolic extracts of *Scrophularia oxystepala* induce apoptosis in MCF-7 breast cancer cells (35) Cervical cancer remains poorly managed by existing anticancer drugs due to limited efficacy and severe side effects. This study identified an active fraction (F2) from dichloromethane extract of *Azadirachta indica* (neem) stem bark with potent antitumor activity against HeLa and ME-180 cells. F2 induced cell cycle arrest, apoptosis, and endoplasmic reticulum (ER) stress while elevating ROS, though apoptosis occurred through ROS-independent ER stress signaling. Importantly, F2 exhibited minimal cytotoxicity toward normal fibroblasts and showed strong growth-inhibitory effects in 3D spheroid tumor models. Phytochemical analysis identified six compounds, including nicotiflorin (a flavonoid) and five limonoids, as key constituents. These findings provide the first evidence of neem stem bark-derived compounds as promising leads for novel cervical cancer therapeutics (36). *Fragaria ananassa* methanolic extract has been shown to upregulate the expression of pro-apoptotic proteins Bax, Bid, and p73 in T-47D cells, while downregulating Bcl-xL expression (1, 37). Additionally, a fraction from *Vitex rotundifolia* leaves significantly induces apoptosis in both MCF-7 and T-47D cells through both extrinsic and intrinsic pathways (38). Similarly, *Viscum album* from *Malus domestica* (VaM) exerts potent anticancer effects against breast cancer by inhibiting cell proliferation, inducing apoptosis, and suppressing STAT3 signaling via SHP-1. VaM synergized with low-dose doxorubicin and significantly reduced tumor growth *in vivo*, supporting its potential as a promising therapeutic for breast cancer (39).

Exposure to methanol and butanol extracts from *Oldenlandia diffusa* (Willd) Roxb. triggered the activation of Caspase-8, -7 in breast cancer (MCF-7) cells, leading to increase in Bax expression and a decrease in Bcl-2 levels (40). In contrast, cell death in MCF-7 cells was inhibited by the chloroform extract of *Cucurbita ficifolia*, which resulted in the upregulation of FADD, BAK, BAX, as well as Caspase-8, -9, and -3 (41). Additionally, treatment of MDA-MB-468 breast cancer cells with extracts from Vatica diospyroides induced apoptosis through the upregulation of Bax (1).

The intrinsic apoptotic pathway is primarily driven by mitochondrial activity within the cell and is regulated by Bcl-2 family protein (42). In contrast, the PI3K/AKT/mTOR signaling pathway promotes cell survival by enhancing the activity of anti-apoptotic factors while suppressing pro-apoptotic factors (9, 42). Baicalin, in combination with doxorubicin, shows potential against doxorubicin-resistant leukemia cells and exhibits apoptosis induction via inhibition of PI3K/AKT signaling pathway and downregulation of antiapoptotic proteins Bcl-2 (43). Resveratrol, a polyphenol found mainly in plant sources such as grapes, blueberries, apple, carrot, peanuts etc. enhance the anti-proliferative effects of chemotherapeutic agents bestatin in chronic myelogenous leukaemia (CML) by decreasing the function and expression of p-glycoprotein (P-gP). It also increases the sensitivity of CML cells toward bestatin induced apoptosis via increasing the activity of apoptosis-related proteins (caspase-3 and 8) and decreasing the phosphorylation of AKT/mTOR (44).

Emodin (6-methyl-1,3,8-trihydroxyanthraquinone), found in variety of plant, fungi and lichen in conjunction with 5-flurouracil (5-FU), has been reported to reverse resistance in colon cancer cells towards 5-FU by downregulating PI3K/AKT pathway and induces apoptosis by decreasing Bcl-2 and increasing caspase-3 and Bax activity (45). Levoshikonin isolated from the root of *Arnebiae Radix* exhibits similar pattern of action thus promoting cell death and arresting cell cycle at G0/G1 in HeLa cell resistant to cisplatin therapy (46). Luteolin, another flavonoid, has been reported (*in vitro and in vivo*) to reverse the resistance in cervical cancer cells to doxorubicin by targeting the PI3K/AKT pathway. Luteolin treatment resulted in upregulated expression of PI3K and caspase-3, however the activity of p-AKT, p-mTOR were found to decreased thereby inhibiting metastasis and inducing apoptosis in cancer cells, making it a valuable therapeutic candidate (9, 47).

Monoterpenes like D-limonene and perillyl alcohol exhibit anticancer effects by inducing apoptosis (1, 48). Taxol, derived from *Taxus brevifolia*, disrupts microtubule dynamics, thereby halting cell division and triggering cell death. In addition this Taxol at higher concentration causes necrosis (1, 12, 49). A study conducted by Mavrogiannis and co-worker explores the effect of Vincaalkaloids such as vincristine and vinblastine on breast cancer cells (BT-20). There was a significant increase in the level of p53 without alteration in Bcl-2 level in BT-20 cells post treatment with Vinca alkaloids. Research has suggested that apoptosis induced by vinca alkaloids follow the mechanism that do not depend on G2/M arrest, which raises the possibility that the apoptosis caused by Vinca alkaloids is not necessarily dependent on cell cycle progression pathway (12, 50). Similarly, in another study (*in vivo*) vincristine administration causes activation of caspase 3/9 in leukemic cells. Lomustine and vincristine, treatment in neuroblastoma cells induces apoptosis and up regulated p21 level. It was established that both these compounds promote apoptosis through the mitochondrial pathway and lead to a decrease in the anti-apoptotic proteins Bcl-xl and Bcl-2 (51–53).

Ouabain, the cardiac glycoside was observed to reduce reactive oxygen species and mitochondrial membrane potential (MMP) levels, while promoting an increase in intracellular Ca2+ levels. It also triggered the activation of pro-apoptotic proteins that led to decrease in anti-apoptotic protein (Bcl-2) expression (54). Oleandrin, a toxic glycoside, induces cell death in human osteosarcoma cells (U2OS and SaOS-2) by triggering apoptosis. This process involves the generation of ROS, which leads to the disruption of mitochondrial membrane potential. Consequently, hydrolytic enzymes are released into the cytosol, and the regulation of certain proteins occurs, including a decrease in Bcl-2 levels and an increase in Bax. Additionally, caspases, specifically caspase-3, -9, and -8, are activated during this apoptotic response (1, 55).

Alkaloids like solamargine, lycorine, and camptothecin exhibit significant anticancer properties (1). Solamargine (SM), a bioactive compound from *Solanum nigrum*, suppressed proliferation and induced apoptosis in renal carcinoma cells (ACHN and 786-O) in a dose- and time-dependent manner. Mechanistically, SM downregulated p-STAT3 and Bcl-2 while upregulating cleaved caspase-3, -8, -9, and Bax, thereby activating apoptotic pathways. *In vivo*, SM inhibited tumor growth in xenografted mice without major organ toxicity, highlighting its therapeutic potential against renal carcinoma (56). Cryptolepine (CRP), an anti-malarial drug, demonstrated selective cytotoxicity against triple-negative breast cancer (TNBC) cells compared to non-TNBC and normal cells. CRP inhibited migration, colony formation, and induced intrinsic apoptosis by reactivating mutant p53 through enhanced DNA binding and transcriptional activation. These findings highlight CRP’s potential as a repurposed therapeutic for TNBC (1, 57). Lycorine, a widely used alkaloid found abundantly in the bulb of *Lycoris radiate (* 58), when given in tongue cancer and liver cancer reduces the level of TCRP1 protein, a factor in chemotherapeutic resistance. This reduction is a result of activation of TCRP1 degradation pathway, which further inhibits the Akt/mTOR pathway ultimately inducing the apoptosis and autophagy pathway (13, 59).

Camptothecin is a natural compound that has very promising anticancer properties and had been previously obtained from the stem and bark of the tree called *Camptotheca acuminata*, also known as happy tree. The primary mechanism of action of camptothecin and its derivatives (trastuzumab, belotecan, irinotecan, topotecan) is through the binding to the DNA as well as topoisomerase II, which together make a ternary complex (60). This interaction greatly impairs the re-ligation of DNA, thereby resulting in damage to DNA and triggering the apoptotic signaling pathway only in cancer cells. The covalent bonding between camptothecin and DNA-topoisomerase is stabilized by hydrogen bonding interactions (12, 60, 61).

The naturally occurring flavonoid dihydromyricetin, derived from the plant *Vitis heyneana* traditionally used in Chinese herbal medicine, induced activation of p53 and apoptosis in ovarian cancer cells resistant to paclitaxel and doxorubicin (62). Apigenin, a widely consumed dietary flavonoid, has been shown to enhance the expression of apaf-1, caspase-8, and p53 level in human CD44+ prostate cancer stem cells. Additionally, apigenin treatment suppresses the expression of Bcl-2, sharpin, and survivin, thereby boosting the efficacy of cisplatin (13, 63, 64). Wogonin is naturally present in various fruits, vegetables, and medicinal plants. It exhibits diverse biological properties, including anti-cancer and anti-inflammatory effects, as well as efficacy against bacterial and viral infections (65). In anti-CD133 human osteosarcoma cancer stem cells, it induces apoptosis by suppressing matrix metalloproteinase-9 expression, thereby reducing cell migration and self-renewal (66, 67). It also promotes ROS accumulation, enhancing TRAIL-induced apoptosis in A549 (13, 67).

Galangin, a flavonoid derived from the root of *Alpinia galanga*, has been shown to effectively inhibit multidrug-resistant cancer cells and exhibits collateral sensitivity, making it a promising therapeutic candidate (68, 69). Research highlights that galangin enhances the p53-dependent apoptotic pathway in ovarian cancer cells, promoting apoptosis more effectively in these resistant cancer cells than in normal ovarian cells (70). Additionally, galangin mitigates cisplatin resistance in lung cancer (A549) cells by suppressing the p-STAT3/p65 and Bcl-2 signaling pathways, further demonstrating its potential as therapeutic agent in resistant cancer (13, 71). The bisbenzyl isoquinoline alkaloids like tetrandrine, derived from Stephania tetrandra, and compounds like oxymatrine and matrine from Sophora flavescens, have demonstrated abilities to reverse multidrug resistance, induce apoptosis, and inhibit proliferation in cancer cells (72).

Curcumin is a bioactive polyphenolic compound derived from the rhizomes of Curcuma longa. Widely recognized for its therapeutic properties, Curcumin is extensively utilized for its antioxidant, anti-inflammatory, wound-healing, and anticancer potential, making it a promising agent for inhibiting cancer initiation and progression (73). Curcumin exerts its anticancer effects by reducing tumor cell proliferation and promoting apoptosis. It achieves this through the upregulation of p53 expression and activity, suppression of anti-apoptotic pathways such as PI3K signaling and MAPKs, and the enhancement of endogenous ROS production. Additionally, Curcumin downregulates anti-apoptotic proteins like Bcl-2 and XIAP, while increasing pro-apoptotic factors such as BAX and BAK (13, 74). Curcumin triggers apoptosis in cancer cells by affecting multiple sites in apoptotic signaling cascades, which increases mitochondrial membrane permeability that allows the release of cytochrome c. Its release further stimulates caspase activation leading to an effective apoptotic response; thus, such the therapeutic potential in cancer treatment might be of multifaceted nature (11, 75).

Despite outstanding therapeutic applications, curcumin faces significant limitations, including poor solubility, limited absorption, restricted tissue distribution, and rapid metabolism. One promising strategy to overcome these challenges is the development of novel curcumin analogs, such as EF24. This analog has been shown to inhibit cancer progression by targeting multiple pathways: suppressing NF-κB and HIF-1α, regulating ROS production, and modulating critical genes through miRNA. These mechanisms collectively arrest the cancer cell cycle and induce apoptosis (76, 77). Additionally, EF24 enhances the sensitivity of cisplatin-resistant ovarian cancer cells by upregulating p53 and p21 proteins at the G2/M checkpoint (13, 78).

β-Elemene (β-ELE), a sesquiterpene compound isolated from Curcuma Rhizoma. Several studies have provided promising therapeutic applications of β-ELE, such as its ability to inhibit cell proliferation, trigger cell cycle arrests, and inducing apoptosis. Its role in reversing multidrug resistance makes it very important for overcoming issues during chemotherapy treatment (79). Studies have demonstrated that exposure of lung cancer cell resistant to cisplatin to β-ELE triggers apoptosis, which is attributed to a decrease in mitochondrial membrane potential and an increase in intracellular ROS level (13, 80). Furthermore, β-ELE may help overcome resistance in gastric cancer by enhancing the expression of Caspase-3, a key protein involved in apoptosis (81). **Table 1** provides an overview of plant-derived natural products as anticancer agents, focusing on their direct or indirect effects on the apoptotic pathway.

**Table 1:** Summary of plant derived natural products as anticancer agent targeting directly or indirectly apoptotic pathway.

Natural products	Structure	Conc. used	Cancer types	Mechanism of action	References
Baicalin	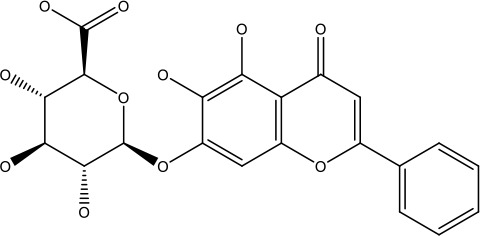	5-10 μM	Leukaemia cell	Causes upregulation of PARP, cleaved caspase 3 and downregulation of MRP1, LRP, Bcl2, p-AKT	(43)
		5-10μg	Liver cancer	Decreases P-lycoprotein and anti-apoptotic Bcl-xlexpression	(82)
		12.5-100 μM	Lung Cancer	Inhibiting of PI3K/Akt/NF- Signaling pathway	(83)
Ursolic acid	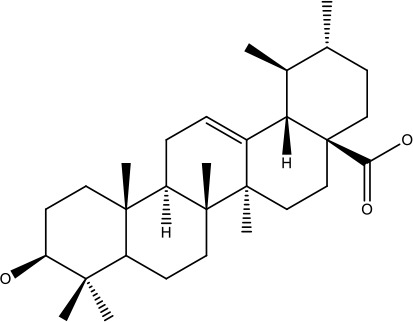	25-500 μM	Lung cancer	Inhibition of mitotic kinase activity	(84)
Apigenin	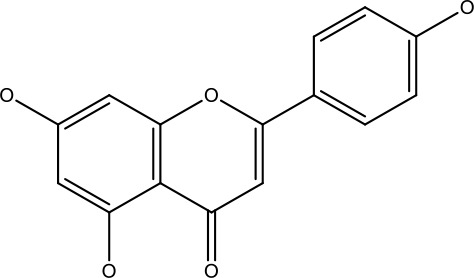	1.56-100 μM	CD44+ prostate cancer stem cells	Resulted in decreased anti-apoptotic Bcl-2, sharping and survivin; and increased proapoptotic caspase-8, Apaf-1 and p53 mRNA	(63)
		5-160 μM	Lung Cancer	Elevate p53 and upregulate certain pro-apoptotic proteins	(85)
		25-100 μM	Colon cancer	Inhibiting ATP-binding cassette (ABC) transporter expression	(86)
Resveratrol	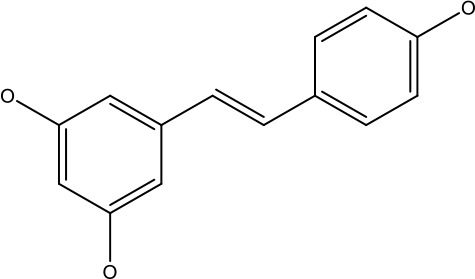	45.9μM	Human leukaemia cancer	Increased caspase 3 and 8 decreased p glycoprotein, MDR1 and p-AKT	(44)
		100-150 μM	Colorectal cancer	Regulate the activity of p53 and inhibits the IGF-IR pathway.	(87)
		160-250 μM	Bladder cancer	Inhibition of the PI3k/Akt/mTOR pathway	(88)
Luteolin	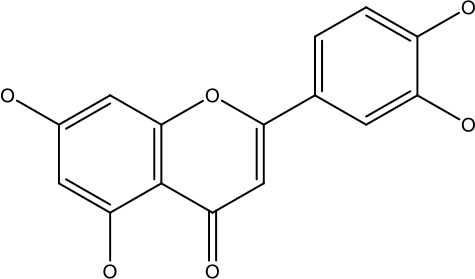	15-30 μM	Glioblastoma	Down regulation of transcription factor NF-κβ leads to DNA breakdown and eventually apoptosis	(12, 89)
		10–200 mg	cervical cancer	Increases the PI3K, Cleavedcaspase 3 level and Decreases p-AKT, p-mTOR, p70S6K, Ki67	(47)
Wogonin	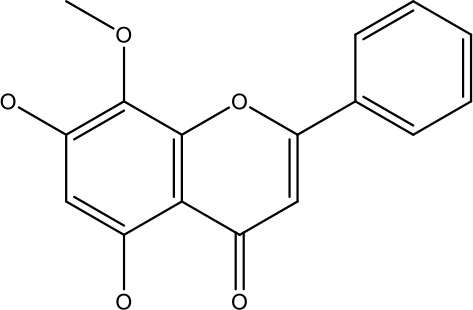	0-200 μM	Colon Cancer	Inhibiting PI3K/Akt signaling pathway	(90)
		20-80 μM		Decreased HIF-1α, HKII, PDHK1, LDHA, PI3K and p-AKT activity.	(91)
Kaempferol	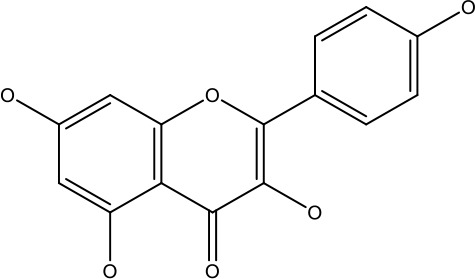	50-300 μM	Chronic myelogenous leukaemia	Sensitises TNF-α related apoptosis inducing ligand resistance and enhance the pro-apoptotic effects of anti-TRAILantibody.	(92)
		15-120μM	Colon Cancer	Inhibited ROS generation; and Modulated the expression of JAK/STAT3,MAPK, PI3K/AKT and NF- κβ	(93)
Safranal	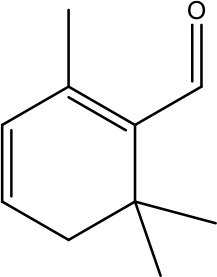	0.8mM	Cervical cancer	Inhibition of malignant cell line proliferation and induces apoptosis	(94)
Galangin	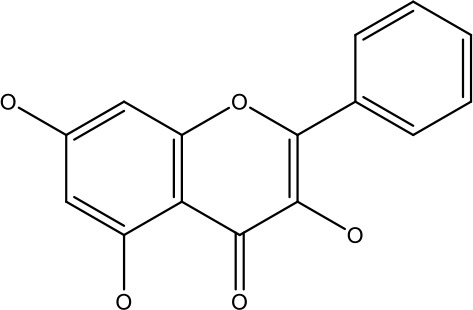	10-40 μM	Ovarian Cancer	Enhancedthe p53-dependent intrinsic and extrinsic apoptotic pathway.	(70)
		0.5-40 μM	Lung Cancer	Inactivating p-STAT3/p65 and Bcl-2 pathways	(71)
Levoshikonin	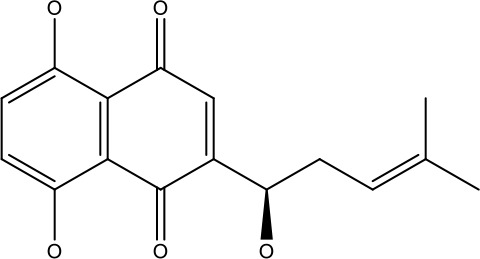	3.92 μM	Cervical cancer	Increases the activity of proapoptotic protein; Bax, Cleaved caspase- 3 and Decreases: Bcl-2	(46)
Emodin	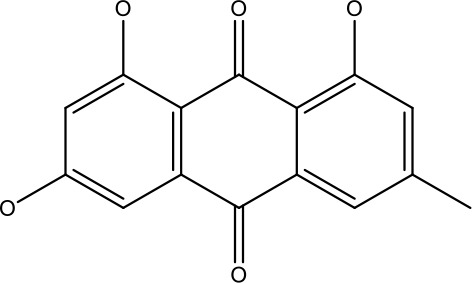	9 μmol	Colorectal cancer	Increases the activity of proapoptotic protein: cleaved caspase-3, Bax and Decreases: Bcl-2, p-ERK, p-AKT	(45)
Curcumin	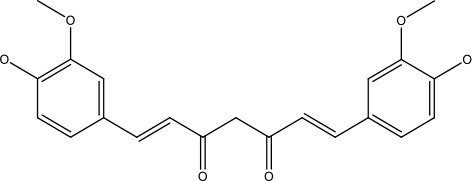	31.62 μmol	Leukaemia cell	Decreases p-glycoprotein, and p-AKT,	(95)
Quercetin	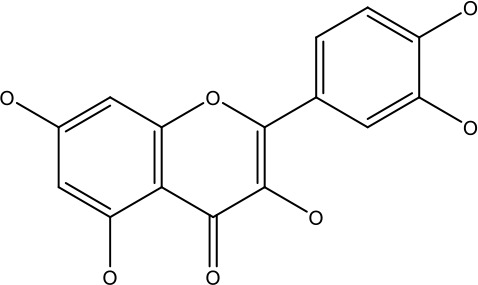	0.005–0.15 μg	Cervical cancer	Decreasesp-AKT, mTOR, p70S6K, and P-gP	(96)
β-elemene	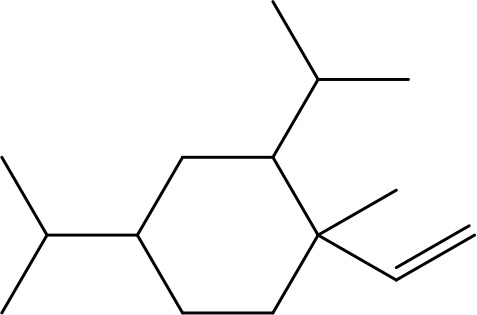	20, 40 μg	Lung cancer	Decreases the MDR1, LRP, p-PI3K, p-AKT	(97)
		5–100 μg/mL	Gastric cancer	Increases the activity of caspase- 3	(98)

## 6 Role of nanoparticle in cancer drug deliver and therapy

Nanotechnology has become an integral part of medicine, finding applications in diagnosis, treatment, and targeted tumor therapy for the improved safety and efficacy of drugs. Advances in cancer nanomedicine have greatly improved the pharmacokinetics such as stability, solubility, circulation time, and bioavailability and pharmacodynamics of anticancer agents (99, 100). Several FDA-approved nanoparticles, including liposomes, albumin nanoparticles, and polymeric micelles, are already being used for cancer treatment, while many other nanocarriers are undergoing clinical trials (101). Nanoparticle (NP)-based drug delivery systems offer significant benefits in cancer therapy, including enhanced pharmacokinetics, precise tumor targeting, reduced side effects, and minimized drug resistance (14, 102). This innovative approach has transformed cancer diagnosis and therapeutic strategies. The perks associated with use of nanomedicine are like enhanced drug bioavailability, selective tumor targeting with minimal off-target cellular uptake, high drug loading capacity. Recent advances in nanotechnology, synthetic biology, and artificial intelligence have led to the development of novel platforms with the potential to revolutionize cancer therapy and overcome drug resistance. There is an increasing demand for low-cost and sustainable natural nanomaterials in cancer theranostics due to their biocompatibility, reduced toxicity, and eco-friendly production processes compared to synthetic alternatives. Naturally derived nanomaterials such as carbon quantum dots, polysaccharide-based nanoparticles, and plant-extract mediated metallic nanoparticles have gained attention for their dual role in diagnosis and therapy, offering targeted drug delivery, imaging capability, and minimal adverse effects (103, 104). Moreover, their synthesis often involves green chemistry approaches, such as using phytochemicals or microbial systems, which significantly lower production costs and environmental impact while ensuring scalability (105). These advantages align with the growing emphasis on sustainable nanomedicine, making natural nanomaterials promising candidates to replace expensive and potentially hazardous synthetic nanocarriers in future cancer management strategies (106, 107).

Stimuli-driven “smart” nanomaterials have shown promise in overcoming MDR by facilitating triggered drug release followed by site-specific accumulation. These nanomaterials are designed to respond to various stimuli, including pH, redox potential, and enzymatic activity. For example, pH-responsive nanoparticles counteract MDR by releasing drugs in the acidic tumor microenvironment, a major contributor to drug resistance in cancer cells (99, 108). The physical properties of nanoparticles (NPs), such as size, shape, and surface characteristics, significantly influence their drug delivery efficiency and therapeutic performance (109). NPs with diameters between 10 and 100 nm are considered optimal for cancer therapy, as they effectively achieve the enhanced permeability and retention (EPR) effect, allowing better drug delivery. Smaller particles (<1–2 nm) risk leaking into normal vasculature and harming healthy cells, while those under 10 nm may be filtered out by the kidneys (14, 110). Conversely, particles larger than 100 nm are prone to clearance by phagocytes (111). Surface characteristics also play a crucial role in determining the bioavailability and circulation half-life of nanoparticles.

### 6.1 Types of nanoparticles in cancer therapy

**Figure 3:**
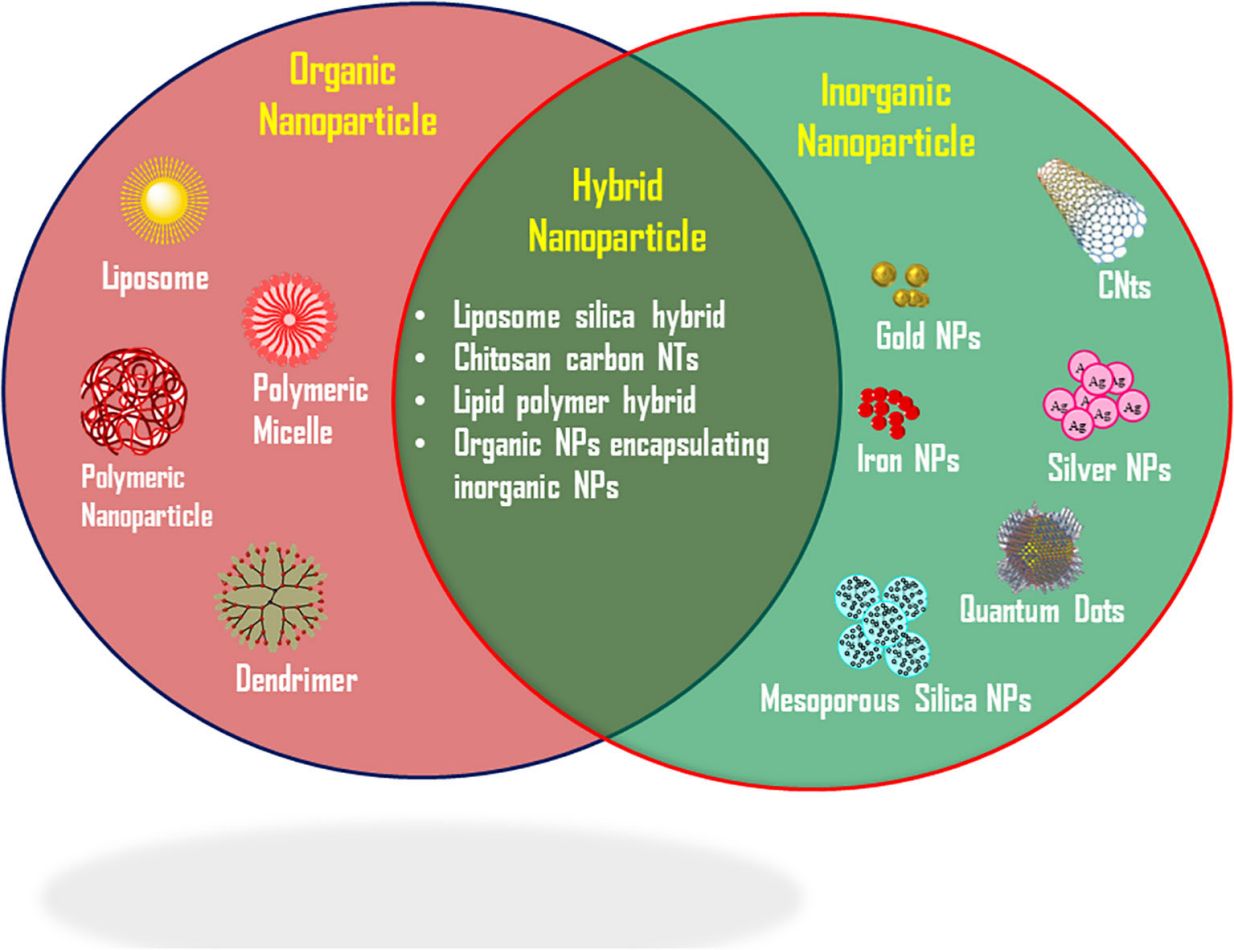
Types of nanoparticles used in cancer therapy.

NPs used extensively in cancer therapy are basically three types Organic NPs, Inorganic NPs and Hybrid NPs (**Figure 3**).

#### 6.1.1 Organic NPs

Organic nanoparticles (NPs) topic of immense attention over a past few decades with an enormous mixture of varied materials. Among them, liposomes were the first nano-drug delivery system approved for clinical use (14, 112). These structures consist of an outer lipid bilayer encapsulating either hydrophobic or hydrophilic drugs (**Figure 3**). By modifying the lipid bilayer, liposomes can mimic biophysical properties such as mobility and deformation characteristic of living cells (113), enabling more efficient therapeutic drug delivery. Liposomes serve as an effective platform for delivering drugs like doxorubicin, paclitaxel, and nucleic acids, showing improved bioavailability and anti-tumor activity (114). FDA approved liposome-based formulations, such as Doxil^®^ and Myocet^®^, of daunorubicin are used to treat metastatic breast cancer (114–116).

Polymeric nanoparticles can be defined as “colloidal macromolecules” with distinct structural configurations formed by various monomers (117). These systems can encapsulate drugs within or attach them to their surface, forming nanocapsules or nanospheres for controlled drug release at specific targets (118). Initially, non-biodegradable polymers like polyacrylamide, polymethylmethacrylate (PMMA), and polystyrene were used to develop polymeric nanoparticle (116, 119). However, their accumulation often resulted in toxicity due to their poor clearance from the body. To address this, biocompatible and biodegradable polymers such as polylactic-co-glycolic acid (PLGA) were introduced. PLGA, a copolymer of glycolic and lactic acids, demonstrates improved compatibility, degradation, and efficacy through the EPR effect, making it a widely used drug delivery carrier (120).

Dendrimers represent another polymer class employed in nanomedicine. These are spherical macromolecules characterized by a well-defined, hyperbranched structure. Other types of organic NPs, including nanoemulsions, extracellular vesicles, solid lipid nanoparticles, and cyclodextrin nanosponges, have also been explored in cancer medicine for applications like drug delivery and stabilization to enhance drug loading capacity (116, 121).

#### 6.1.2 Inorganic NPs

Inorganic nanoparticles (NPs) offer the advantage of a high surface-area-to-volume ratio, enabling their various biomedical uses. Among these, extensively studied types include gold NPs (AuNPs), silver NPs (AgNPs), carbon nanotubes (CNTs), quantum dots, magnetic nanoparticles (MNPs), and silica nanoparticles (SNPs) (**Figure 3**). Carbon nanotubes, discovered in the late 1980s, are cylindrical structures often viewed as rolled graphene sheets. They are classified into two main types: single-walled and multi-walled. Their carbon-based nature enables interactions with immune cells, potentially triggering immune responses that suppress tumor growth. Due to their unique physical, chemical, and biological properties, CNTs have significant potential in drug delivery. They have been employed to deliver anticancer agents such as doxorubicin, paclitaxel, methotrexate, and siRNA for various cancers (14, 122). Their rolled graphene structure provides a large surface area, extended length, and narrow diameter, allowing for efficient therapeutic molecule loading. Furthermore, the oxygen-containing functional groups on their surfaces facilitate surface modifications, enabling ligand-targeted approaches (123).

Quantum dots are semiconductors characterized by broad absorption spectra, narrow emission bands, and exceptional photostability. These properties make them highly useful in biological imaging (116, 124). Beyond imaging, quantum dots are being explored for cancer therapy, with graphene quantum dots being particularly noteworthy due to their biocompatibility and rapid excretion. Magnetic nanoparticles, which typically contain metals or metal oxides, are widely applied in MRI imaging and drug delivery. Coating these NPs with polymers or fatty acids enhances their stability and biocompatibility (125). They have demonstrated considerable efficacy in chemotherapy and gene therapy for cancer treatment (14, 126).

Metallic nanoparticles, including AuNPs, AgNPs, iron-based NPs, and CuNPs, are prominent in biological imaging and targeted drug delivery due to their magnetic, optical, and photothermal properties. Gold nanoparticles, in particular, are extensively studied. Functionalized AuNPs enhance drug accumulation in tumors and can overcome drug resistance due to their inert, non-toxic gold cores (127, 128, 129). They possess unique features such as a large surface area, the ability to conjugate with various molecules, excellent biocompatibility, and controlled drug-release capabilities (14).

Silica nanoparticles, especially mesoporous silica nanoparticles (MSNs), are highly suitable for drug delivery (**Figure 3**). Their large internal pore volume allows them to encapsulate significant quantities of anticancer drugs, while supramolecular caps enable controlled drug capture and release (123, 124). Due to their superior pharmacokinetics, high stability, and enhanced treatment efficacy, silica nanoparticles are considered among the most effective drug delivery vehicles (130, 131).

#### 6.1.3 Hybrid NPs

It is quite evident that both organic and inorganic nanoparticles (NPs) have distinct strengths and limitations (14, 116). The hybridization of these two types of differing NPs in the same drug delivery system develops a multifunctional platform, which offers significantly improved biological properties (**Figure 3**) (116). Such new strategy not only enhances the therapeutic efficacy of drugs, but it also plays a crucial role to tackle the long-standing problem of drug resistance. A notable approach involves hybridizing natural biomaterials with organic or inorganic NPs to optimize their design (132). For instance, liposome-silica hybrid nanoparticles, comprising a silica core encased by a lipid bilayer, have been successfully synthesized and validated for delivering drugs to effectively target prostate and breast cancer cells (133). Additionally, liposome silica hybrids NPs have demonstrated potential as a synergistic delivery platform for gemcitabine and paclitaxel in treating pancreatic cancer in a mouse model (134). Lipid-polymer hybrid NPs, featuring a polymeric core surrounded by a lipid shell, have emerged as a promising drug delivery system for managing various cancers, including pancreatic cancer (135), breast cancer (136), and metastatic prostate cancer (137). These hybrid nanoparticles leverage the high biocompatibility of lipids alongside the robust structural integrity of polymer NPs, enabling the encapsulation of both hydrophilic and hydrophobic drugs. This dual encapsulation capability contributes to enhanced therapeutic outcomes (14, 116, 138).

### 6.2 AI/ML in NP design

Artificial intelligence (AI), particularly machine learning (ML), is reshaping nanoparticle research by enabling precise prediction, synthesis optimization, and autonomous design. ML models can infer critical physicochemical properties such as size, shape, surface chemistry, and optical behavior from synthesis parameters like precursor concentration, temperature, pH, and reaction time, thus minimizing costly trial-and-error experiments (107, 134). For example, random forest and neural network models have been successfully applied to predict the morphology of gold and silver nanoparticles, while Bayesian optimization has been used to tune perovskite nanocrystals for desired optical properties with fewer experimental trials (105). Furthermore, deep learning–based inverse design strategies have facilitated the creation of plasmonic core–shell nanoparticles with tailored scattering and absorption properties, applications that would otherwise be prohibitively time-consuming using conventional methods (135). Such approaches not only accelerate discovery but also support environmentally sustainable and resource-efficient synthesis strategies.

Beyond materials design, AI plays a pivotal role in advancing nanoparticle-based drug delivery systems by enhancing therapeutic precision and safety. By integrating large datasets of nanoparticle traits, ML algorithms can optimize parameters such as size, surface charge, and functionalization for improved targeting, controlled release, and minimized toxicity (106). Practical examples include the optimization of polymeric nanoparticles for doxorubicin delivery, where surface properties were fine-tuned for tumor-specific accumulation, and the application of deep learning models to predict the biodistribution of lipid nanoparticles used in mRNA vaccines, enabling refinement of lipid compositions for higher efficiency. AI-driven frameworks are also modeling nanoparticle pharmacokinetics, predicting clearance and tissue distribution through multimodal deep learning, thereby promoting safer and more predictable therapies. In addition, smart nanocarriers equipped with biosensors have been designed to respond to biological cues such as pH-sensitive nanoparticles releasing drugs in acidic tumor microenvironments demonstrating enhanced treatment outcomes (136). Collectively, these applications illustrate how AI and ML guide the rational design of next-generation nanoparticles for biomedical, industrial, and environmental applications.

## 7 Phytochemical based nanomedicine targeting apoptotic for treatment of cancer resistance

Phytochemicals have demonstrated significant potential as anticancer agents in preclinical studies across various cancer types (1, 4, 13, 14). However, their clinical application is hindered by inherent limitations, including poor water solubility, low bioavailability, instability, and short circulation time. To address these challenges, researchers are increasingly focusing on developing phytochemical-based nanoformulations, known as phytonanomedicine. These nanoformulations enhance the solubility of phytochemicals in aqueous environments, improve pharmacokinetics by maintaining steady therapeutic levels over extended periods, and enable targeted delivery to tumors with increased cellular uptake (4, 14, 99, 116).

Plant extracts play a vital role in the eco-friendly synthesis of nanoparticles (NPs). They not only serve as stabilizing and capping agents but also act as reducing agents during NP synthesis. This green synthesis approach utilizes the interaction of phytochemicals with specific metals or metal oxides, such as gold, copper, silver, iron, and titanium, to produce NPs. The resulting plant-derived metal nanoparticles have garnered substantial scientific interest due to their nontoxicity, eco-friendliness, cost efficiency, and enhanced optical properties. Furthermore, these biocompatible plant-based nanoparticles outperform chemically synthesized counterparts in terms of safety and application (12, 139–141).

Nimbolide, a terpenoid extracted from Azadirachta indica, has gained substantial recognition over the past decade for its robust and multifaceted anti-cancer properties. A recent study introduced a PLGA nanoformulation encapsulating nimbolide and assessed its therapeutic potential through *in vitro* and in silico methods. The findings revealed that nimbolide-loaded nanoparticles effectively suppressed cell proliferation and promoted mesenchymal-to-epithelial transition by concurrently inhibiting AKT and mTOR signaling pathways, showing superior efficacy compared to nimbolide only. This mesenchymal-to-epithelial transition activation further induced apoptosis, enhanced chemosensitivity, and diminished tumor-initiating potential in pancreatic cancer stem cells (116, 136). PLGA nanoparticles loaded with apigenin (101.3 ± 0.004 nm) have demonstrated significant anti-cancer activity by restricting skin tumor progression in mice models subjected to benzo[a]pyrene and UV-B exposure. This effect was attributed to the suppression of epidermal hyperplasia and modulation of key biomarkers associated with mitochondrial apoptosis, cell proliferation, and intracellular ROS levels (99, 139, 142). In another investigation, intravenous administration of liquid crystalline nanoparticles co-delivering pemetrexed and resveratrol resulted in notable tumor growth suppression in urethane-induced lung cancer in mice. The mechanism involved the inhibition of angiogenesis and the induction of apoptosis (143). Similarly, quercetin encapsulated in methoxy-PLA nanoparticles exhibited significant tumor growth reduction in female BALB/c xenograft models. TUNEL assays indicated a higher incidence of apoptosis in tumors treated with encapsulated quercetin compared to quercetin only at equivalent doses (99, 144). Furthermore, resveratrol encapsulated in bovine serum albumin nanoparticles was effective in reducing tumor growth in ovarian carcinoma in nude mice by activating the mitochondrial apoptotic pathway (145).

Gold nanoparticle (AuNPs) exhibits numerous advantages that make them highly suitable for cancer therapy. Their small size allows them to penetrate cells effectively, while their EPR effect facilitates build-up at tumor sites (146). Additionally, AuNPs, due to their high atomic number absorb X-rays more efficiently than conventional agents, providing superior imaging contrast. Upon exposure to light of specific energy, AuNPs resonate to produce heat, which can be harnessed for photothermal therapy of tumor cells (12, 147).

Plant-based methods for synthesizing AuNPs have demonstrated significant potential. For instance, *Zataria multiflora* leaf extract mediated synthesis of AuNPs exhibits antiproliferative effects against human cervical cancer (HeLa) cells (148). These AuNPs inhibited cell proliferation and triggered apoptosis in a dose-dependent pattern by activating caspase-3 and -9. Similarly, rhizome extract of *Curcuma wenyujin* has been utilized to produce AuNPs, which exhibited apoptosis-inducing effects in human renal carcinoma (A498) cells, with a cytotoxic concentration (CC50) value of 25 g/mL (149). These AuNPs caused mitochondrial membrane damage, nuclear damage, and ROS generation, culminating in apoptosis. Furthermore, green-synthesized AuNPs have been shown to exert dose-dependent toxicity against A549 cells, suppressing cell growth, activating caspases, and downregulating anti-apoptotic proteins. The ethanolic extracts of *Syzygium aromaticum* were employed for green synthesis of AuNPs, which effectively reduced the viability of human malignant lymphoma (SU-DHL-4) cells, with enhanced ROS generation and apoptosis in a time- and dose-dependent manner (12, 150). The aqueous leaf extract of *Alternanthera sessilis* has also served as a reducing agent for AuNPs synthesis, showing cytotoxicity against cervical cancer cells (HeLa). Additionally, ethanolic extract of Siberian ginseng has been used to synthesize AuNPs. It promotes apoptosis in melanoma (B16) cells. This observed result was achieved by reducing mitochondrial membrane potential, increasing ROS levels in mitochondria, upregulating pro-apoptotic genes (Bad, Bid, Caspase-9, and Caspase-3), and downregulating the anti-apoptotic Bcl2 gene (151).

Silver nanoparticles (AgNPs) have emerged as promising candidates for cancer therapy due to their nanoscale size and their ability to induce cell death through mechanisms such as oxidative stress, chromosomal instability, and ds DNA break (152). Studies have demonstrated the anticancer and apoptotic effects of AgNPs synthesized using *Fagonia indica*, which effectively inhibit the growth of MCF-7 breast cancer cells in a concentration-dependent manner, with an IC50 value of 12.35µg/mL. This effect is primarily mediated by the ROS generation, leading to oxidative stress, nuclear condensation, and cell membrane damage, ultimately resulting in cell death (153).

Similarly, AgNPs synthesized from *Beta vulgaris* root extracts have shown cytotoxic effects on normal (CHANG) and cancerous hepatic cells (HuH-7). The findings indicated that these NPs generate significantly higher ROS levels in HuH-7 cells compared to normal cells, inducing DNA strand breaks and ultimately enhancing apoptosis (12, 154). AgNPs synthesized using *Ginkgo biloba* leaf extract have demonstrated anticancer activity against cervical cancer cells by inhibiting cell proliferation and triggering apoptosis through elevated intracellular ROS levels. The treatment also activates the caspase-dependent mitochondrial apoptotic pathway in these cells (155). Moreover, AgNPs derived from *Sargassum vulgare* alginate extracts have shown selective toxicity towards HL60 and HeLa cancer cell lines. In another study, AgNPs synthesized from tamarind fruit shells were found to induce apoptosis in human breast cancer cells by causing DNA damage and disrupting mitochondrial electron transport. These AgNPs exhibit dose-dependent anticancer effects, further emphasizing their potential in targeted cancer therapy (156).

Zinc oxide NPs (ZnO NPs) have demonstrated significant potential as drug carriers, effectively delivering therapeutic agents to targeted sites (12, 157). Their ability to induce ROS generation and promote apoptosis makes ZnO NPs as promising candidates for anticancer therapy (158). Additionally, the nano-sized structure of ZnO enhances zinc bioavailability within the body. Compared to other metal oxide nanoparticles, ZnO NPs are notably less toxic and more cost-effective, making them suitable for various therapeutic applications, particularly in cancer (159).

ZnO NPs synthesized using *Rehmanniae radix* have been evaluated for their anticancer activity against osteosarcoma cell (MG-63) (160). Studies revealed that these green-synthesized NPs effectively inhibited MG-63 cell proliferation at higher doses, increased ROS production, decreased mitochondrial membrane potential, and upregulated pro-apoptotic proteins such as Bax, caspase-3, and caspase-9. Similarly, ZnO NPs biosynthesized from *Marsdenia tenacissima* have shown significant anticancer activity against the Hep-2 laryngeal cancer cells (12, 161). The findings demonstrated their cytotoxicity and pro-apoptotic effects on Hep-2 cells, as evidenced by enhanced ROS generation, nuclear damage, and MMP disruption. Apoptotic potential was further confirmed by the downregulation of the anti-apoptotic protein Bcl-2 and the upregulation of pro-apoptotic proteins like Bax, caspase-9, and caspase-3. Moreover, ZnO NPs synthesized using the leaf extract of *Solanum nigrum* were investigated for their antiproliferative efficacy against cervical cancer (HeLa cell lines) (162). The results highlighted a dose-dependent cytotoxicity, where ZnO NPs inhibited β-catenin expression and elevated levels of caspase-3, caspase-9, and p53, leading to apoptosis. The anticancer effects of ZnO NPs derived from *Marsdenia tenacissima* against Hep-2 cells were also confirmed, reiterating their ability to induce DNA damage, ROS production, disrupt MMP, and modulate apoptotic proteins (161).

Compared to other metal nanoparticles, copper nanoparticles (CuNPs) are notable for their ease of synthesis, which can be achieved through simple, cost-effective methods that yield high production rates (12, 163). CuNPs prepared using *Quisqualis indica* flower extract has demonstrated significant cytotoxic and apoptotic effects on melanoma cells (164). These effects are attributed to mechanisms such as ROS generation, LDH release, and the reduction of intracellular glutathione levels. Likewise, CuNPs derived from *Phaseolus vulgaris* exhibit apoptosis-inducing and growth-inhibitory effects on cervical cancer cells (165). In another study, CuNPs prepared using *Olea europaea* leaf extract showed dose-dependent cytotoxic effect through promoting apoptosis in breast (AMJ-13) and ovarian (SKOV-3) cancer cells (166). Additionally, CuNPs derived from *Ziziphus zizyphus* leaf extract were tested on human renal carcinoma cells, revealing significant cytotoxicity characterized by increased ROS production and apoptosis. This apoptotic effect was confirmed by the upregulation of pro-apoptotic markers like Bid, Bax, and caspases-3 and -9, along with the downregulation of the anti-apoptotic gene Bcl-2 (167). The *Azadirachta indica* leaf extract derived CuONPs has also been explored for its cytotoxic activity against MCF-7 and HeLa cells (168). Treatment with these CuONPs resulted in induced cell toxicity, ROS generation, and DNA break in cancer cells. Moreover, there was a notable increase in apoptotic protein markers, including Bax, caspase-9, caspase-8, caspase-3, P38, and cytochrome c, was observed in cancer cells (12, 168).

Currently, there are no plant-based nanoparticle formulations approved by the U.S. Food and Drug Administration (FDA) for cancer therapy. While these NPs have not yet entered large-scale clinical trials, some have reached early-stage clinical testing, primarily as part of experimental studies. Only a limited number of nano-enabled phytochemical formulations have advanced to clinical evaluation in oncology, with nanocurcumin standing out as the most clinically advanced candidate. For instance, an oral nanocurcumin formulation (NCT02724618) is currently being assessed in a Phase II trial as a radioprotective adjuvant during prostate radiotherapy, aiming to mitigate treatment-related toxicities (169). This highlights both the potential and the rarity of such approaches, as the majority of ongoing phytochemical-based studies in cancer still focus on non-nano supplements or prevention-oriented interventions, rather than therapeutic applications in drug-resistant cancers.

### 7.1 Bio-safety and economic aspects of plant-based nanoparticles/extract:

The biosafety of plant-based nanoparticles in cancer treatment is a critical consideration, particularly given the complex biological environments they interact with. Recent studies have demonstrated that plant-based nanoparticles generally exhibit superior biocompatibility and reduced systemic toxicity compared to chemically synthesized nanoparticles, largely due to the natural origin of their capping and stabilizing phytochemicals (170). These phytochemicals not only contribute to the therapeutic effect but also minimize inflammatory and immune responses. Furthermore, *in vivo* studies have shown that these nanopartcles can selectively accumulate in tumor tissues through the enhanced permeability and retention effect, thereby reducing off-target effects and improving therapeutic index (171). For instance, green-synthesized gold and silver nanoparticles using Withania somnifera or Azadirachta indica extracts have demonstrated potent anticancer activity with minimal cytotoxicity to normal cells (172). Moreover, their ability to overcome drug resistance by modulating signaling pathways and drug efflux mechanisms has been highlighted in recent cancer models (173). Nonetheless, long-term toxicity, pharmacokinetics, and biodistribution profiles remain underexplored, and further comprehensive *in vivo* and clinical studies are essential to fully validate their biosafety for translational use.

Beyond their therapeutic promise, the economic aspects of plant-derived nanoparticles and natural products are crucial for their translation into clinical practice. Conventional anticancer therapies, particularly synthetic chemotherapeutics and monoclonal antibodies, often involve high manufacturing costs, complex purification steps, and sophisticated cold-chain requirements, which collectively limit accessibility in low- and middle-income countries (174, 175). In contrast, phytochemical-based nanoparticles are generally more cost-effective due to the abundance, renewability, and relatively simple extraction of plant materials, along with eco-friendly synthesis processes that reduce production expenses (176).

Moreover, green synthesis of nanoparticles using plant extracts eliminates the need for expensive toxic chemicals and energy-intensive procedures, making it a sustainable and economically viable alternative (177). Importantly, plant-based compounds and their nanoformulations also demonstrate potential for scalability and integration into existing pharmaceutical frameworks, which can further reduce costs associated with large-scale manufacturing (178).

Economic evaluations have also highlighted that natural product-based interventions could lower the overall burden of cancer care by reducing side effects, minimizing hospitalizations, and improving patient compliance (179). Thus, the incorporation of cost-effectiveness assessments into future preclinical and clinical studies will be vital for strengthening the case for large-scale adoption of these therapies in oncology (180).

### 7.2 Phytochemical based nanomedicine for treatment of cancer resistance: advantages, challenges and alternative approaches

#### 7.2.1 Advantage associated with phytochemical based nanomedicine in cancer treatment

1. Improved pharmacokinetics and tumor targeting: NP delivery markedly improves solubility, circulation time, stability and tumor accumulation via the EPR effect, increasing phytochemical bioavailability and therapeutic index. This enhances apoptosis induction and can reverse multidrug resistance when used alone or in combination with chemotherapeutics (4).
2. Controlled and stimuli-responsive release: “Smart” NPs (pH/redox/enzyme responsive) enable site-specific drug release in the acidic/reductive tumor microenvironment, reducing systemic toxicity and improving intratumor drug concentrations (10, 11).
3. Synergy & combination delivery: Hybrid lipid-polymer or liposome-silica platforms allow co-delivery (e.g., phytochemical + chemo agent or siRNA) to produce synergistic anticancer effects and overcome resistance (101).
4. Diagnostic/theranostic utility: Certain inorganic NPs (Au, Ag, quantum dots, magnetic NPs) provide strong imaging contrast (CT, optical, MRI) or photothermal/photodynamic capabilities enabling simultaneous detection and treatment (theranostics). This improves cancer detection sensitivity and allows image-guided therapy (142).
5. Eco-friendly production and reduced hazardous reagents: plant extracts can act as reducing/stabilizing agents in NP synthesis, yielding biocompatible, lower-cost products with attractive optical/biological properties relative to harsh chemical routes (142).

#### 7.2.2 Challenges

Despite the promising potential of plant-based nanoparticles and natural products in overcoming cancer drug resistance, several significant challenges need to be addressed for their effective clinical translation. One major challenge is the inconsistency in the quality and purity of plant-based materials used for nanoparticle synthesis. The variability in plant extracts due to geographical, seasonal, and genetic factors can lead to inconsistencies in nanoparticle size, shape, and surface properties, which may affect their pharmacological activity and biosafety (170). Furthermore, large-scale production of plant-based nanoparticles remains a hurdle, as scalable synthesis methods that ensure batch-to-batch consistency are not yet standardized (181).

Another challenge is the limited understanding of the precise mechanisms through which these phytochemical loaded nanoparticles modulate apoptotic pathways in resistant cancer cells. While *in vitro* studies have demonstrated promising anticancer effects, the translation of these findings into *in vivo* models remains difficult due to issues related to nanoparticle bio-distribution, accumulation, and clearance (182). Additionally, while these nanoparticles are often considered biocompatible, their long-term toxicity and accumulation in organs like the liver, spleen, and kidneys must be thoroughly investigated to rule out potential side effects and off-target toxicity (171).

Drug resistance is another critical issue in the clinical application of plant-based nanoparticles. While these nanoparticles can potentially reverse MDR by modulating drug efflux pumps and apoptotic pathways, their efficacy can be reduced due to the complex heterogeneity of tumor cells and the emergence of resistance mechanisms at the cellular and molecular levels (183). Furthermore, the challenge of overcoming the blood-brain barrier for brain cancer treatments adds another layer of complexity to the effective use of plant-based nanoparticles in treating resistant cancers (184).

#### 7.2.3 Alternative approaches to overcome these challenges

To address the challenge of variability in plant extract quality, researchers are focusing on standardization and optimization of the extraction processes. Advanced techniques such as green synthesis using specific plant parts (e.g., leaves, seeds, or roots) and the use of high-throughput screening for quality control can help ensure batch consistency and reproducibility of plant-based nanoparticles (185). Additionally, the use of molecular docking and *in silico* methods to better predict the interaction between phytochemicals and cancer targets could help in designing nanoparticles with enhanced therapeutic efficacy and specificity (186).

In terms of improving bio-distribution and minimizing toxicity, recent developments in nanoparticle surface modification, such as functionalization with targeting ligands (e.g., antibodies, peptides, or aptamers), have shown promise in enhancing tumor-specific accumulation and reducing off-target effects (187). For example, conjugating plant-based nanoparticles with folate or transferrin has been shown to improve their uptake by cancer cells overexpressing folate receptors or transferrin receptors (188). Additionally, the incorporation of stimuli-responsive materials (e.g., pH-sensitive or enzyme-sensitive coatings) allows for controlled drug release specifically at the tumor site, reducing systemic toxicity (182).

To combat MDR, combination therapies involving these NPs with conventional chemotherapeutic agents or immune modulators could enhance therapeutic outcomes. For instance, combining plant-based nanoparticles with chemotherapeutic drugs like cisplatin or paclitaxel has been shown to synergistically overcome MDR by inhibiting drug efflux pumps and restoring apoptotic pathways (184). Furthermore, the use of nanocarriers capable of co-delivering genetic material, such as siRNA or miRNA, alongside natural products could help target key signaling pathways involved in drug resistance (183).

Lastly, overcoming the blood-brain barrier for the treatment of brain cancers can be facilitated by modifying plant-based nanoparticles with specific ligands targeting blood-brain barrier receptors, such as the transferrin receptor or low-density lipoprotein receptor (187). Additionally, the use of nanoparticle formulations that can undergo passive diffusion across the blood-brain barrier or apply ultrasound-mediated drug delivery are emerging strategies to enhance therapeutic efficacy in brain tumors (186).

## 8 Conclusion

Cancer remains a complex, multi-step disease and a major global health burden, where conventional treatments such as chemotherapy often fail due to primary or acquired resistance and adverse side effects. Tumors acquire drug resistance through diverse mechanisms, including drug efflux, DNA repair, epithelial–mesenchymal transition, alterations in the tumor microenvironment, and evasion of apoptosis. Epigenetic modifications and genetic mutations further contribute to multidrug resistance (MDR). Since apoptosis is central to maintaining normal cellular homeostasis, targeting dysregulated apoptotic pathways presents an effective strategy to combat both cancer progression and MDR.

Plant-based natural products have emerged as promising alternatives owing to their abundance, low toxicity, and broad bioactivities. However, challenges such as poor solubility, bioavailability, and stability limit their clinical use. Phytonanomedicine offers solutions by enhancing pharmacokinetics, enabling tumor-specific delivery through the enhanced permeability and retention (EPR) effect, and improving therapeutic efficiency. Natural compounds and plant-derived nanoparticles have shown potential to regulate MDR-related genes, inhibit PI3K/AKT/mTOR signaling, and induce apoptosis, autophagy, and cell cycle arrest.

Looking ahead, future research must focus on unraveling molecular mechanisms of phytochemicals and nanocarriers, particularly their interactions with resistance-related proteins like P-glycoprotein and Bcl-2 family members. Incorporating multi-omics approaches could help identify patient-specific biomarkers to guide precision therapies. Combination strategies that integrate phytochemicals with conventional drugs or immunotherapies may further enhance efficacy, as seen with curcumin analogs and β-elemene in resistant cancer models.

Nanomedicine represents a transformative approach, especially with smart, stimuli-responsive and hybrid nanoparticles capable of controlled release, tumor targeting, and real-time imaging. Green synthesis of nanoparticles using plant extracts also provides a cost-effective, eco-friendly, and safer alternative for clinical development. At the same time, translational challenges must be addressed, including formulation standardization, regulatory validation, long-term toxicity studies, and improved preclinical models such as organoids and patient-derived xenografts.

In conclusion, plant-based natural products and phytonanomedicine hold strong promise to redefine cancer therapy by overcoming MDR through apoptosis modulation. With interdisciplinary research and clinically driven innovation, these strategies could pave the way for personalized, safer, and more effective cancer treatments in the near future.
